# NoSAS score and the 5-year risk of incident cerebrovascular disease: a retrospective cohort study

**DOI:** 10.3389/fmed.2026.1756799

**Published:** 2026-03-03

**Authors:** Huimin Chen, Xiaomi Chen, Junqi Ren, Shuyue Zhou, Huan Li, Zhaojun Chen, Yihuan Su, Dongjie Huang, Siyu He, Xinyao Liu, Tingting Sun, Qinghua Chen, Enlin Ye, Junfen Cheng, Baozhi Zhang, Riken Chen, Lijuan Zeng, Yuli Cai

**Affiliations:** The Second Affiliated Hospital of Guangdong Medical University, Zhanjiang, Guangdong, China

**Keywords:** a retrospective cohort study, cerebrovascular disease, NoSAS questionnaire, obstructive sleep apnea (OSA), prevalence

## Abstract

**Objectives:**

Obstructive sleep apnea (OSA) is a common but often underdiagnosed disorder linked to various adverse health outcomes, especially cerebrovascular events. This study aims to evaluate the utility of the NoSAS score in screening for high risk of cerebrovascular disease, facilitating early identification and improving clinical management of OSA in susceptible populations.

**Methods:**

This retrospective study enrolled patients from two sleep centers who underwent sleep apnea monitoring between September 2016 and December 2019. Individuals with pre-existing cerebrovascular disease were excluded from the study. Follow-up data on 5-year incident cerebrovascular events were collected via medical records or telephone interviews. Cerebrovascular events were primarily identified based on patient self-report and available clinical documentation; systematic neuroimaging confirmation was not routinely available. Participants were categorized by NoSAS score into high-risk (≥8) and low-risk (<8) groups. Logistic regression was used to assess the association between NoSAS risk classification and cerebrovascular disease incidence, and Nelson-Aalen were used to compare compared cumulative risk between groups over the 5-year period.

**Results:**

A total of 1,348 participants with complete NoSAS score data who completed follow-up were included in the analysis. Among the 690 participants in the high-risk group, the 5-year incidence of cerebrovascular disease was 9.71%. In the adjusted model, the NoSAS high-risk group had a 1.8-fold increased risk of cerebrovascular disease compared to the low-risk group (OR: 1.85, 95% CI: 1.01∼3.38; *p* = 0.045). ESS-stratified analyses showed significant associations between NoSAS risk classifications and cerebrovascular disease in ESS scores ≤ 9.

**Conclusion:**

The NoSAS high-risk group showed a higher incidence of cerebrovascular disease, which could be used as an independent predictor of the disease and may have higher predictive value in the high-risk group of non-sleepy OSA.

## 1 Background

Obstructive sleep apnea (OSA) is a syndrome of chronic intermittent hypoxia, carbon dioxide retention(when present), recurrent microarousals, and structural sleep abnormalities result from repeated partial or complete collapse of the upper airway during sleep (1). OSA is one of the most common sleep disorders, affecting approximately 30% of the population in Europe and the United States. In Asia, patients with moderate to severe OSA account for about 20% of adult males and 10% of postmenopausal females (2). OSA may increase the risk of cardiovascular and metabolic diseases such as hypertension, coronary heart disease, cardiac arrhythmia, type 2 diabetes, and stroke by 2–3 times (3–6). Patients with untreated OSA are more likely to develop other conditions, including cardiovascular disease, post-operative complications, and road traffic accidents (7–9).

Cerebrovascular disease, including stroke, transient ischemic attack, and other vascular disorders affecting the brain, is the most common and serious form of cerebrovascular disease, the leading cause of disability and the sixth leading cause of death in the United States (10). According to estimates by the American Heart Association, the direct and indirect costs of stroke in the United States exceeded $51.2 billion in 2003, representing a significant cost to the economy (11). It is projected that by 2030, 3.8% of U.S. adults will have experienced a stroke, and annual direct medical costs due to stroke are expected to increase from $71.55 billion to $181.3 billion (12). As the population ages, the incidence and disease burden of cerebrovascular disease is expected to increase, highlighting the importance of preventing the occurrence of cerebrovascular disease.

The severity of OSA can be determined by the AHI, which is categorized as no OSA [apnea hypopnea index (AHI) < 5], mild OSA (AHI 5–15), moderate OSA (AHI 5–15), and severe OSA (AHI ≥ 30) (13). In ischemic stroke patients, the proportions of mild, moderate, and severe sleep apnea were 72, 63, and 38%, respectively, and the combination of OSA increased the recurrence rate of stroke and morbidity and mortality (14, 15). Several large epidemiological studies abroad have shown that these two diseases are highly correlated and that OSAS is an independent risk factor for CVD (16). Therefore, early diagnosis and treatment of OSA is very important to prevent complications and may also help prevent the incidence and recurrence of stroke.

Polysomnogram (PSG) is currently used as the gold standard for the diagnosis of OSA. However, this test is time-consuming, expensive and complex and requires experienced technicians. In addition, patients are required to wait a long time for the completion of a sleep study, which greatly limits the widespread use of these diagnostic tests in suspected OSA cases (17). Therefore, a simple, easy-to-operate, and economical screening questionnaire is needed to screen eligible patients. To this end, many clinical prediction models have been developed based on clinical, demographic, and anthropometric variables (18, 19). The NoSAS score is a new screening tool developed and validated in recent years in a population-based study. The NoSAS score ranges from 0 to 17 and is assigned for 5 different items (20). When scores reach 8, they perform significantly better than the STOP-Bang and Berlin questionnaires and have a larger area under the ROC curve (AUC), suggesting that the NoSAS score is a valid tool for identifying individuals at high risk for sleep apnea (21).

In conclusion, OSA is the most common sleep disorder and can also put many chronic diseases at increased risk. However, OSA has a high rate of underdiagnosis, and the NoSAS scale developed in recent years for OSA screening has improved the detection rate of OSA. However, no study has yet investigated the clinical value of the NoSAS score in the prediction of cerebrovascular disease in patients with OSA, and the aim of this study was to investigate the predictive ability of the NoSAS score for cerebrovascular disease in patients with OSA.

## 2 Materials and methods

### 2.1 Data sources and participants

**FIGURE 1:**
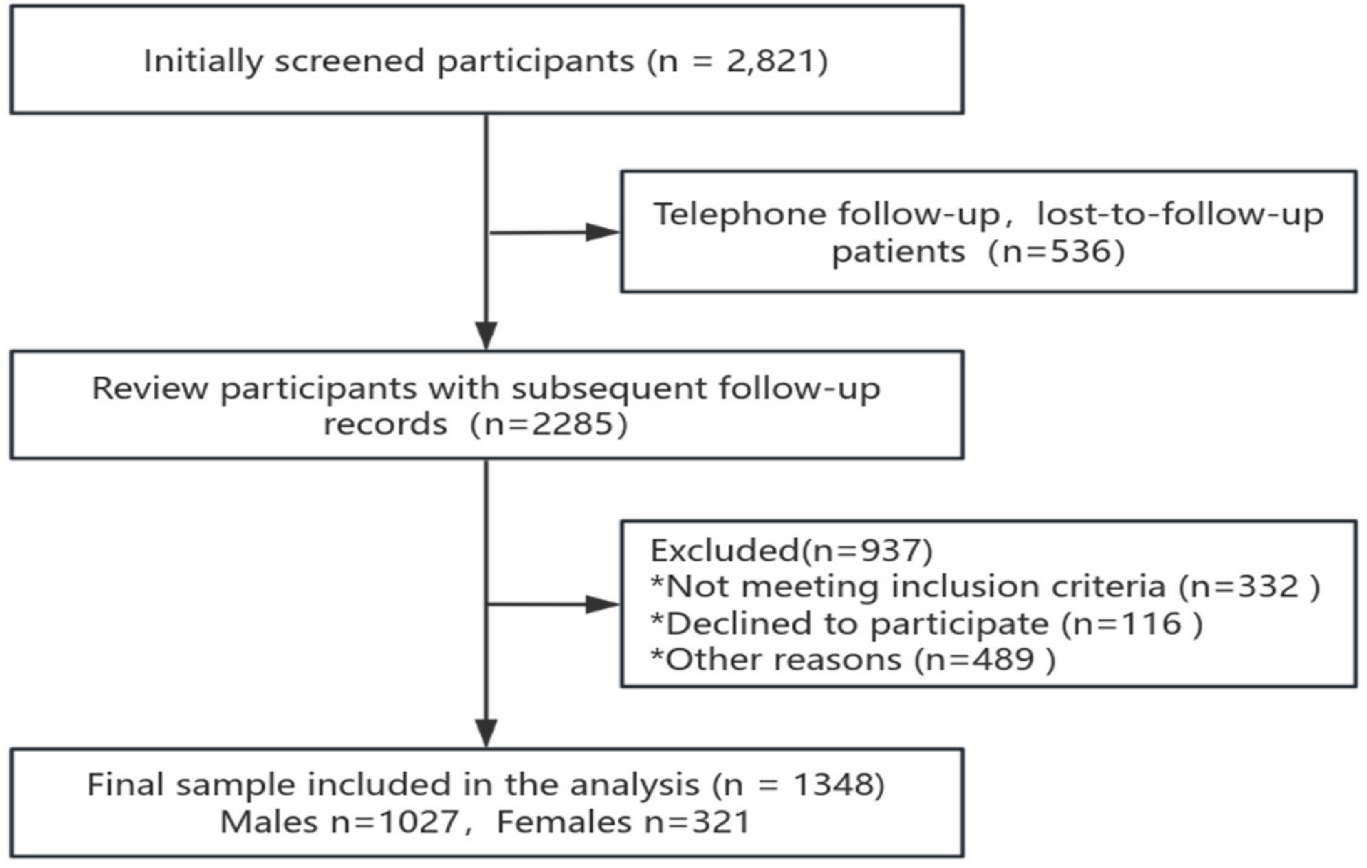
Participant inclusion flowchart.

This study was a retrospective, prospective cohort study. Participants were primarily patients referred to the sleep clinic due to symptoms suggestive of sleep-disordered breathing, including habitual snoring, nocturnal breathing pauses, and excessive daytime sleepiness. In addition, some participants were referred following routine health examinations or high-risk screening. All enrolled participants underwent preliminary evaluation by sleep specialists and were included in the study only if they met the suspected sleep-disordered breathing criteria. Participants were included from patients who underwent sleep apnea monitoring at the Sleep Medicine Centre of the First Affiliated Hospital of Guangzhou Medical University and the Institute of Sleep Diseases of the Second Affiliated Hospital of Guangdong Medical University from 1 September 2016 to 31 December 2019, and the inclusion process is shown in Figure 1. Ethical approval for the study was obtained from the Ethics Committee of the First Affiliated Hospital of Guangzhou Medical University (Ethics No. 2022183) and the Ethics Committee of the Second Affiliated Hospital of Guangdong Medical University (Ethics No. PJKT2024-050). All participants signed an informed consent form. In this study, follow-up was conducted through a combination of medical record system data collection and telephone follow-up. Cerebrovascular events were primarily identified based on patient self-report and available clinical documentation; systematic neuroimaging confirmation was not routinely available. The research team first conducted medical record system data collection for patients enrolled in the study after the completion of 5 years to obtain information about the patients’ new-onset cerebrovascular diseases. For patients whose information was not recorded in the medical record system, the team conducted additional data collection through telephone follow-up. During the telephone follow-up, the researchers will ask the patients in detail whether they have had a cerebrovascular event in the past 5 years and record the exact time of the event. This type of follow-up, which combines data collection from the medical record system and telephone follow-up, aims to ensure the completeness and accuracy of the data, thus providing reliable support for the study results.

Inclusion criteria required participants to meet the following four criteria: (1) age 18 years or older; (2) ability to complete the questionnaire; and (3) capacity for autonomous behavior and awareness. Exclusion criteria included any participant who (1) had a pre-existing diagnosis of cerebrovascular disease prior to admission or at baseline; (2) had a clear diagnosis of another serious neurological disorder (e.g., Parkinson’s, epilepsy) at baseline state; (3) was on long-term or persistent use of sedatives or sleeping pills; (4) had severe organ failure that prevented them from completing the test; and (5) had incomplete questionnaire responses.

### 2.2 NoSAS scores

The NoSAS score is a simple, validated tool for screening for OSA, developed by Marti-Soler et al. at the University of Lausanne, Switzerland (20). The NoSAS score ranges from 0 to 17 and is assigned as follows: Neck circumference of more than 40 cm is scored as 4 points; a BMI between 25 and 30 kg/m^2^ is scored as 3 points or a BMI at or above 30 kg/m^2^ scored 5 points; snoring scored 2 points; age over 55 years scored 4 points; and male scored 2 points. In this study, those with a score of 8 and above were categorized as high risk, while those with a score of less than 8 were categorized as low risk. The main covariates in this study included gender, age, BMI, neck circumference, systolic blood pressure, diastolic blood pressure, smoking status, alcohol consumption and diabetes status.

### 2.3 Diagnostic criteria

Cerebrovascular diseases refer to a variety of disorders affecting the cerebral vasculature and cerebral circulation, including those that may lead to acute disruption of the cerebral circulation and subsequent acute neuronal injury and those that may lead to chronic pathological changes and neurological deficits in the small vessels (22). In this study, cerebrovascular disease was primarily determined based on patient self-report and available clinical documentation. Follow-up telephone interviews were conducted to ascertain whether participants had ever been diagnosed with cerebrovascular disease by a physician and to record the approximate date of onset.

### 2.4 Statistical analysis

Continuous variables with normal distribution were expressed as mean [standard deviation (SD)] and independent samples *t*-test was used to assess between-group differences. Categorical variables, on the other hand, were expressed as percentages, and chi-square tests were used to assess between-group differences. To examine whether the NoSAS high-risk group independently predicted the incidence of cerebrovascular disease, variables related to cerebrovascular disease were corrected in the unadjusted model. Multivariate analyses were used to correct for variables that showed statistical significance in the unadjusted analyses. Nelson-Aalen cumulative risk function plots show the cumulative risk of cerebrovascular disease over a 5-year period for participants in the NoSAS high-risk group and the low-scoring group. Stratified analyses were also performed according to sex, age, and ESS score (ESS score above 9 indicates excessive daytime sleepiness). All tests were two-tailed, and statistical significance was set at *p* < 0.05. Statistical analyses were performed using IBM SPSS Statistics for Windows version 25.0 (IBM Corporation, Armonk, NY, United States).

## 3 Results

### 3.1 Clinical characteristics of the high-risk and low-risk groups

**FIGURE 2:**
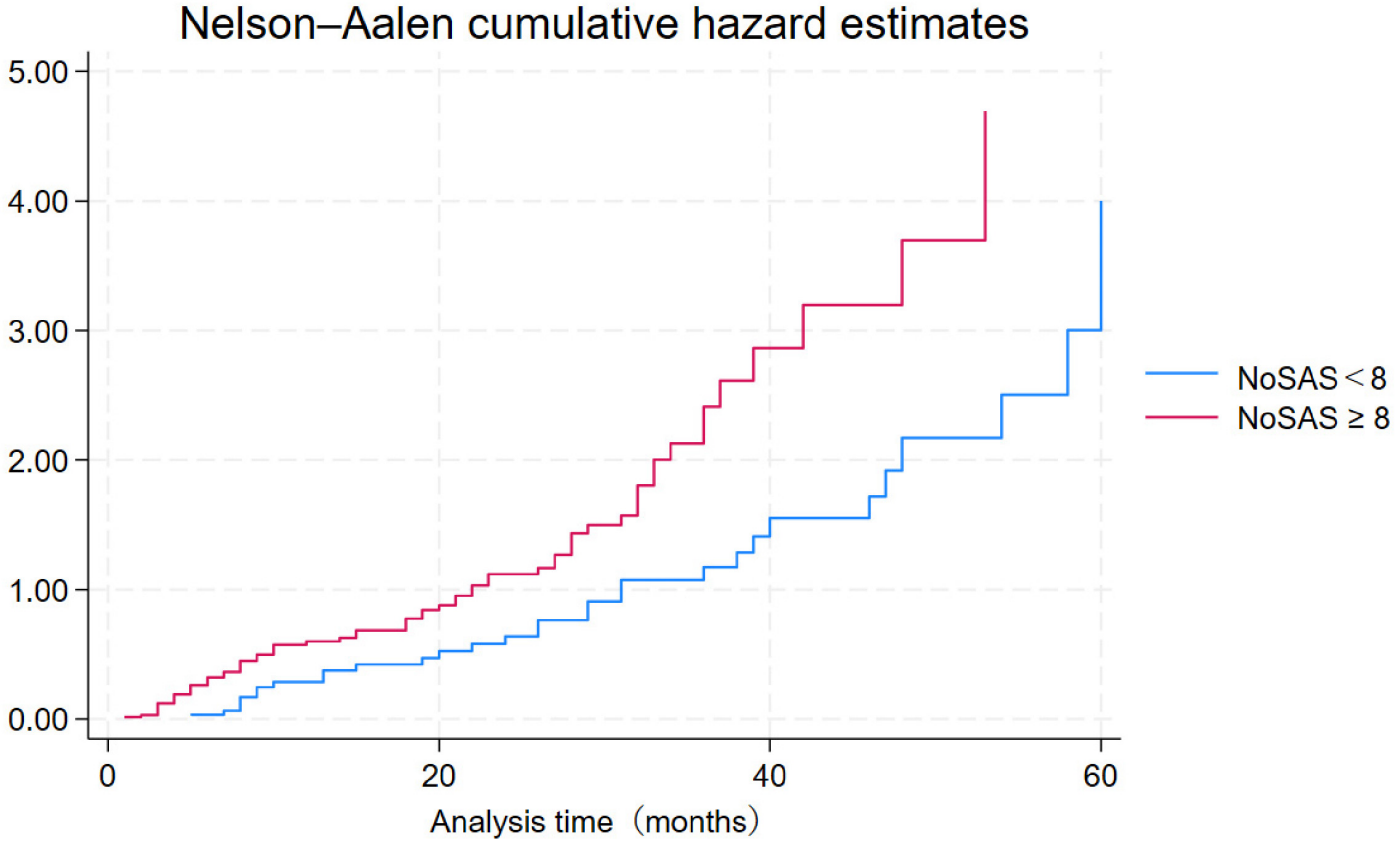
Cumulative risk of cerebrovascular disease over 5 years.

A final total of 1348 participants provided complete NoSAS questionnaire data and access to follow-up information (Table 1). The low-risk group included 658 participants with a 4.71% incidence of cerebrovascular disease, whereas the high-risk group included 690 participants with a 9.71% incidence. Baseline demographic characteristics of the low-risk and high-risk groups are summarized in Table 1. **As** expected from the NoSAS scoring criteria, the high-risk group had a higher proportion of males and higher mean values for age, BMI, neck circumference, systolic blood pressure, diastolic blood pressure, and heart rate than the low-risk group. The cumulative risk of cerebrovascular disease over 5 years was significantly higher in the high- and low-risk groups compared with the low-risk group (Figure 2).

**TABLE 1:** Baseline characteristics.

Variables	Total (*n* = 1,348)	NoSAS low-risk group (*n* = 658)	No SAS high-risk group (*n* = 690)	Statistic	*P*
Prevalence, (%)	98 (7.27)	31 (4.71)	67 (9.71)		
Age (years), n (%)	46.95 ± 13.78	44.31 ± 13.50	49.47 ± 13.58	χ^2^= 36.93	**< 0.001**
<60	1102 (81.75)	581 (88.30)	521 (75.51)		
≥ 60	246 (18.25)	77 (11.70)	169 (24.49)		
Gender, n (%)				χ^2^= 116.33	**< 0.001**
Male	1027 (76.19)	417 (63.37)	610 (88.41)		
Female	321 (23.81)	241 (36.63)	80 (11.59)		
BMI (kg/cm^2^)	26.12 ± 4.13	24.21 ± 3.43	27.94 ± 3.92	t = –18.64	**< 0.001**
Neck circumference (cm)	37.87 ± 4.13	35.54 ± 3.05	40.09 ± 3.78	t = –24.37	**< 0.001**
Systolic blood pressure (mmHg)	128.50 ± 16.85	125.83 ± 17.02	131.05 ± 16.29	t = –5.75	**< 0.001**
Diastolic blood pressure (mmHg)	78.45 ± 12.51	76.54 ± 12.02	80.28 ± 12.69	t = –5.54	**< 0.001**
Heart rate (bpm)	80.79 ± 13.23	79.89 ± 13.04	81.66 ± 13.37	t = –2.43	**0.015**
Smoking, n (%)				χ^2^= 45.54	**< 0.001**
No	868 (64.39)	483 (73.40)	385 (55.80)		
Yes	480 (35.61)	175 (26.60)	305 (44.20)		
Alcohol drinking, n (%)				χ^2^= 14.69	**< 0.001**
No	1033 (76.63)	534 (81.16)	499 (72.32)		
Yes	315 (23.37)	124 (18.84)	191 (27.68)		
Hypertension, n (%)				χ^2^= 43.85	**< 0.001**
No	997 (73.96)	540 (82.07)	457 (66.23)		
Yes	351 (26.04)	118 (17.93)	233 (33.77)		
Diabetes, n (%)				χ^2^= 16.69	**< 0.001**
No	1256 (93.18)	632 (96.05)	624 (90.43)		
Yes	92 (6.82)	26 (3.95)	66 (9.57)		

### 3.2 Relationship between NoSAS risk stratification and the incidence of cerebrovascular disease

In unadjusted analyses, factors associated with the incidence of cerebrovascular disease included age, neck circumference, BMI, systolic blood pressure, smoking, alcohol consumption, hypertension, and diabetes (Table 2). In addition, those in the high-risk group demonstrated significant associations with cerebrovascular disease incidence compared with those in the low-risk group. After adjusting for significant variables in unadjusted analyses, NoSAS risk stratification remained a significant predictor of increased cerebrovascular disease incidence. The risk was 1.8 times higher in the high-risk group than in the low-risk group (OR: 1.85, 95% CI: 1.01∼3.38; *p* = 0.045).

**TABLE 2:** Risk of cerebrovascular disease in all study participants.

Variables	Unadjusted model					adjusted model				
	β	S.E	*Z*	*P*	OR (95% CI)	β	S.E	*Z*	*P*	OR (95% CI)
Age, ≥ 60	2.56	0.23	11.05	**< 0.001**	12.93 (8.21∼20.37)	1.98	0.27	7.41	**< 0.001**	7.24 (4.29∼12.22)
Gender, Female	–0.15	0.25	–0.58	0.565	0.86 (0.52∼1.42)					
Neck circumference	–0.05	0.02	–2.07	**0.038**	0.95 (0.91∼0.99)	0.02	0.04	0.70	0.486	1.03 (0.96∼1.10)
BMI	–0.10	0.03	–3.91	**< 0.001**	0.90 (0.85∼0.95)	–0.13	0.04	–3.29	**< 0.001**	0.88 (0.82∼0.95)
Systolic blood pressure	0.02	0.01	2.87	**0.004**	1.02 (1.01∼1.03)	0.00	0.01	0.17	0.869	1.00 (0.99∼1.02)
Diastolic blood pressure	–0.01	0.01	–0.61	0.543	0.99 (0.98∼1.01)					
Heart rate	–0.01	0.01	–1.06	0.291	0.99 (0.97∼1.01)					
Smoking	–0.51	0.24	–2.15	**0.032**	0.60 (0.37∼0.96)	–0.34	0.29	–1.17	0.243	0.71 (0.40∼1.26)
Drinking	–0.73	0.30	–2.41	**0.016**	0.48 (0.26∼0.87)	–0.16	0.36	–0.44	0.657	0.85 (0.42∼1.73)
Hypertension	1.51	0.22	7.01	**< 0.001**	4.52 (2.96∼6.89)	1.17	0.25	4.65	**< 0.001**	3.21 (1.96∼5.25)
Diabetes	1.51	0.27	5.50	**< 0.001**	4.53 (2.64∼7.76)	0.85	0.32	2.63	**0.009**	2.35 (1.24∼4.44)
**NoSAS group**										
Low-risk					1.00 (Reference)					1.00 (Reference)
High-risk	0.78	0.22	3.46	**< 0.001**	2.18 (1.40∼3.38)	0.62	0.31	2.01	**0.045**	1.85 (1.01∼3.38)

### 3.3 NoSAS risk stratification and cerebrovascular disease prevalence, stratified by sex

In sex-stratified analyses, unadjusted model analyses for both men and women showed a significant association between the NoSAS high-risk group and the incidence of cerebrovascular disease; however, none of the adjusted analyses showed a significant association between NoSAS risk stratification and the incidence of cerebrovascular disease (Tables 3, 4).

**TABLE 3:** Risk of cerebrovascular disease incidence in men.

Variables	Unadjusted model					Adjusted model				
	β	S.E	*Z*	*P*	OR (95% CI)	β	S.E	*Z*	*P*	OR (95% CI)
Age, ≥ 60	2.52	0.26	9.88	**< 0.001**	12.47 (7.56∼20.58)	2.01	0.32	6.26	**< 0.001**	7.44 (3.97∼13.95)
Neck circumference	–0.10	0.03	–3.22	**0.001**	0.91 (0.85∼0.96)	–0.05	0.04	–1.33	0.182	0.95 (0.88∼1.02)
BMI	–0.12	0.03	–3.76	**< 0.001**	0.89 (0.83∼0.94)	–0.07	0.05	–1.46	0.143	0.94 (0.86∼1.02)
Systolic blood pressure	0.02	0.01	2.47	**0.014**	1.02 (1.01∼1.03)	0.00	0.01	0.06	0.950	1.00 (0.98∼1.02)
Diastolic blood pressure	–0.01	0.01	–0.84	0.402	0.99 (0.97∼1.01)					
Heart rate	–0.01	0.01	–1.13	0.261	0.99 (0.96∼1.01)					
Smoking	–0.59	0.25	–2.35	**0.019**	0.55 (0.34∼0.91)	–0.51	0.30	–1.72	0.086	0.60 (0.33∼1.08)
Drinking	–0.78	0.31	–2.49	**0.013**	0.46 (0.25∼0.85)	–0.17	0.36	–0.46	0.645	0.85 (0.41∼1.73)
Hypertension	1.49	0.24	6.14	**< 0.001**	4.43 (2.75∼7.12)	1.39	0.29	4.76	**< .001**	4.02 (2.27∼7.11)
Diabetes	1.70	0.31	5.57	**< 0.001**	5.49 (3.01∼10.00)	1.11	0.37	3.01	**0.003**	3.03 (1.47∼6.24)
**NoSAS group**										
Low-risk					1.00 (Reference)					1.00 (Reference)
High-risk	0.72	0.27	2.67	**0.008**	2.05 (1.21∼3.46)	0.11	0.36	0.30	0.765	1.11 (0.55∼2.25)

**TABLE 4:** Risk of cerebrovascular disease incidence in women.

Variables	Unadjusted model					Adjusted model				
	β	S.E	*Z*	*P*	OR (95% CI)	β	S.E	*Z*	*P*	OR (95% CI)
Age, ≥ 60	4.13	1.03	4.00	**< 0.001**	62.19 (8.20∼471.46)	3.87	1.04	3.72	**< 0.001**	47.94 (6.24∼368.19)
Neck circumference	0.04	0.07	0.66	0.507	1.04 (0.92∼1.19)					
BMI	–0.08	0.05	–1.53	0.127	0.92 (0.83∼1.02)					
Systolic blood pressure	0.02	0.01	1.41	0.157	1.02 (0.99∼1.04)					
Diastolic blood pressure	0.00	0.02	0.14	0.886	1.00 (0.97∼1.04)					
Heart rate	–0.00	0.03	–0.19	0.848	1.00 (0.95∼1.05)					
Smoking	0.10	1.06	0.09	0.926	1.10 (0.14∼8.87)					
Drinking	0.27	1.07	0.26	0.799	1.31 (0.16∼10.69)					
Hypertension	1.65	0.48	3.44	**< 0.001**	5.23 (2.04∼13.41)	1.07	0.52	2.06	**0.040**	2.92 (1.05∼8.11)
Diabetes	0.79	0.66	1.20	0.231	2.21 (0.60∼8.13)					
**NoSAS group**										
Low-risk					1.00 (Reference)					1.00 (Reference)
High-risk	1.09	0.46	2.39	**0.017**	2.99 (1.22∼7.33)	0.37	0.51	0.72	0.470	1.45 (0.53∼3.94)

### 3.4 Age-stratified NoSAS risk stratification and cerebrovascular disease incidence rates

In age-stratified analyses, both NoSAS risk groups and cerebrovascular disease prevalence did not reach statistical significance (Tables 5, 6).

**TABLE 5:** Risk of cerebrovascular disease incidence in subjects aged < 60 years.

Variables	Unadjusted model					Adjusted model				
	β	S.E	*Z*	*P*	OR (95% CI)	β	S.E	*Z*	*P*	OR (95% CI)
Gender, Female	–2.09	1.02	–2.05	**0.041**	0.12 (0.02∼0.91)	–2.53	1.05	–2.42	**0.016**	0.08 (0.01∼0.62)
Neck circumference	0.04	0.05	0.86	0.391	1.04 (0.95∼1.14)					
BMI	0.00	0.04	0.06	0.954	1.00 (0.92∼1.09)					
Systolic blood pressure	0.01	0.01	1.17	0.241	1.01 (0.99∼1.03)					
Diastolic blood pressure	0.01	0.01	0.52	0.602	1.01 (0.98∼1.04)					
Heart rate	–0.03	0.02	–1.77	0.076	0.97 (0.94∼1.00)					
Smoking	0.07	0.37	0.18	0.860	1.07 (0.51∼2.22)					
Drinking	–0.15	0.44	–0.36	0.722	0.86 (0.37∼2.01)					
Hypertension	1.91	0.38	4.98	**< 0.001**	6.72 (3.18∼14.23)	1.83	0.40	4.61	**< 0.001**	6.21 (2.86∼13.51)
Diabetes	2.41	0.41	5.82	**< 0.001**	11.11 (4.94∼25.02)	2.32	0.45	5.18	**< 0.001**	10.20 (4.24∼24.54)
**NoSAS group**										
Low-risk					1.00 (Reference)					
High-risk	0.73	0.38	1.91	0.056	2.07 (0.98∼4.36)					

**TABLE 6:** Risk of cerebrovascular disease incidence in subjects aged ≥ 60.

Variables	Unadjusted model					Adjusted model				
	β	S.E	*Z*	*P*	OR (95% CI)	β	S.E	*Z*	*P*	OR (95% CI)
Gender, Female	–0.48	0.31	–1.57	0.117	0.62 (0.34∼1.13)					
Neck circumference	–0.01	0.03	–0.29	0.775	0.99 (0.93∼1.05)					
BMI	–0.08	0.04	–2.26	**0.024**	0.92 (0.86∼0.99)	–0.10	0.04	–2.47	**0.014**	0.91 (0.84∼0.98)
Systolic blood pressure	0.00	0.01	0.32	0.752	1.00 (0.99∼1.02)					
Diastolic blood pressure	–0.00	0.01	–0.12	0.908	1.00 (0.97∼1.02)					
Heart rate	0.01	0.01	0.75	0.452	1.01 (0.98∼1.04)					
Smoking	–0.66	0.35	–1.90	0.057	0.52 (0.26∼1.02)					
Drinking	–0.72	0.47	–1.52	0.129	0.49 (0.19∼1.23)					
Hypertension	0.75	0.29	2.57	**0.010**	2.11 (1.20∼3.73)	0.82	0.30	2.76	**0.006**	2.27 (1.27∼4.07)
Diabetes	0.10	0.39	0.26	0.797	1.11 (0.51∼2.38)					
**NoSAS group**										
Low-risk					1.00 (Reference)					
High-risk	0.09	0.31	0.30	0.764	1.10 (0.60∼2.02)					

### 3.5 NoSAS risk stratification and cerebrovascular disease incidence stratified by ESS score

Among participants with ESS scores below 9, the NoSAS high-risk group was significantly associated with cerebrovascular disease incidence (Table 7). Among participants with ESS scores above 9, unadjusted model analyses showed that the NoSAS high-risk group was significantly associated with cerebrovascular disease incidence; however, adjusted analyses did not show a significant association between NoSAS risk stratification and cerebrovascular disease incidence (Table 8).

**TABLE 7:** Risk of cerebrovascular disease incidence in patients with ESS ≤ 9.

Variables	Unadjusted model					Adjusted model				
	β	S.E	*Z*	*P*	OR (95% CI)	β	S.E	*Z*	*P*	OR (95% CI)
Age, ≥ 60	2.68	0.29	9.35	**< 0.001**	14.58 (8.32∼25.57)	2.05	0.31	6.54	**< 0.001**	7.78 (4.21∼14.40)
Gender, Female	-0.38	0.31	-1.21	0.225	0.69 (0.37∼1.26)					
Neck Circumference	–0.06	0.03	–2.20	**0.028**	0.94 (0.89∼0.99)	0.01	0.04	0.33	0.739	1.01 (0.94∼1.10)
BMI	–0.16	0.03	–4.78	**< 0.001**	0.85 (0.80∼0.91)	–0.20	0.05	–4.17	**< 0.001**	0.82 (0.75∼0.90)
Systolic Blood Pressure	0.02	0.01	2.91	**0.004**	1.02 (1.01∼1.04)	0.01	0.01	0.67	0.500	1.01 (0.99∼1.02)
Diastolic Blood Pressure	–0.00	0.01	–0.05	0.963	1.00 (0.98∼1.02)					
Heart Rate	–0.00	0.01	–0.33	0.739	1.00 (0.97∼1.02)					
Smoking	–0.51	0.29	–1.75	0.080	0.60 (0.34∼1.06)					
Drinking	–0.74	0.39	–1.91	0.056	0.48 (0.22∼1.02)					
Hypertension	1.43	0.26	5.60	**< 0.001**	4.17 (2.53∼6.88)	1.08	0.31	3.52	**< 0.001**	2.93 (1.61∼5.33)
Diabetes	1.52	0.34	4.47	**< 0.001**	4.57 (2.35∼8.91)	0.90	0.41	2.20	**0.028**	2.46 (1.10∼5.47)
**NoSAS group**										
Low-risk					1.00 (Reference)					1.00 (Reference)
High-risk	0.69	0.26	2.69	**0.007**	2.00 (1.21∼3.31)	0.83	0.35	2.36	**0.018**	2.29 (1.15∼4.54)

**TABLE 8:** Risk of cerebrovascular disease incidence in people with ESS > 9.

Variables	Unadjusted model					Adjusted model				
	β	S.E	*Z*	*P*	OR (95% CI)	β	S.E	*Z*	*P*	OR (95% CI)
Age, ≥ 60	2.25	0.41	5.53	**< 0.001**	9.47 (4.27∼21.00)	1.79	0.44	4.03	**< 0.001**	6.01 (2.51∼14.36)
Gender, Female	0.31	0.45	0.68	0.494	1.36 (0.56∼3.30)					
Neck Circumference	0.01	0.05	0.13	0.900	1.01 (0.92∼1.11)					
BMI	0.03	0.05	0.71	0.475	1.03 (0.94∼1.13)					
Systolic blood pressure	0.01	0.01	0.77	0.442	1.01 (0.99∼1.03)					
Diastolic blood pressure	–0.02	0.02	–1.02	0.308	0.98 (0.95∼1.01)					
Heart rate	–0.02	0.02	–1.21	0.227	0.98 (0.95∼1.01)					
Smoking	–0.47	0.43	–1.11	0.269	0.62 (0.27∼1.44)					
Drinking	–0.62	0.50	–1.24	0.215	0.54 (0.20∼1.44)					
Hypertension	1.81	0.42	4.32	**< 0.001**	6.09 (2.69∼13.82)	1.45	0.44	3.28	**0.001**	4.26 (1.79∼10.12)
Diabetes	1.57	0.47	3.31	**< 0.001**	4.81 (1.90∼12.18)	0.94	0.53	1.78	0.075	2.56 (0.91∼7.24)
**NoSAS group**										
Low-risk					1.00 (Reference)					1.00 (Reference)
High-risk	1.27	0.50	2.54	**0.011**	3.57 (1.34∼9.56)	0.45	0.55	0.81	0.416	1.56 (0.53∼4.58)

## 4 Discussion

This study provides new and comprehensive information on the predictive ability of NoSAS to detect cerebrovascular disease in OSA patients. First, the NoSAS high-risk group had a 1.8-fold higher risk of cerebrovascular disease than the low-risk group. Stratified by ESS score, NoSAS risk stratification with ESS scores less than 9 and cerebrovascular disease incidence showed significant associations. However, neither stratification by gender nor age reached statistical significance.

Due to the high prevalence of undiagnosed OSA and its complications, a reliable screening tool is essential for the timely prediction of OSA (23). To this end, various questionnaires have been used to date. The NoSAS scoring system was initially developed in a population-based study as a simple and validated test for OSA screening. The NoSAS questionnaire contains fewer items than the Berlin, ESS, and STOP-Bang questionnaires. The NoSAS scoring system contains five scoring components, contains more objective biometric items than the BQ, and contains only one subjective factor (snoring) (24). Moreover, age, gender, BMI, and neck circumference variables in the NoSAS questionnaire can be easily and accurately determined, so that this diagnostic tool can be used effectively and conveniently by clinicians and participants. In recent years, a growing number of findings have demonstrated the superiority of the NoSAS score over the Berlin, STOP-Bang, and STOP scores in the diagnosis of OSA (25–27). The sensitivity of the NoSAS score increases with increasing AHI (28, 29).

Bassetti et al.’s study first confirmed the relationship between OSA and stroke (30). A new observational cohort study of 1,022 patients at Yale University in the United States found that OSA was significantly associated with stroke and death, with a hazard ratio of 2.24 (95% CI: 1.30–3.86, *P* = -0.0041); after correcting for the influences of age, sex, smoking, alcohol consumption, BMI, lipids, diabetes mellitus, hypertension, and atrial fibrillation, the hazard ratio was still 1.97 (95% CI: 1.12–3.48, *P* = 0.01), further suggesting that OSA is an independent risk factor for (31). The biological mechanisms between OSA and cerebrovascular disease are mainly (1) Reduced cerebral blood flow in patients with OSA: The application of inhalation of 32Xe (xenon)-labeled single-photon emission computed tomography (SPECT) revealed that OSA patients Cerebral blood flow in gray matter areas was significantly reduced and correlated with the duration of apnea and the degree of oxygen desaturation (32). (2) Increased sympathetic activity significantly increases the risk of hypertension and cerebrovascular disease. Somers (33) found that the termination of respiratory events was accompanied by an arousal response. At the same time, blood pressure rises, heart rate increases, and sympathetic activity is enhanced. The increased sympathetic nerve activity leads to insulin resistance as well as lipid metabolism disorders in the body, both of which are involved in the development of cerebral atherosclerosis, which in turn leads to cerebrovascular disease. Liu et al. (34) further confirmed that hyperlipidemia occurs in OSAS through the action of insulin resistance, and is not a result of OSAS *per se*. Isobe et al. (35) showed that hypoxia may cause insulin resistance. (3) Intermittent hypoxia and recurrent hypoxia during sleep occur with pathological changes similar to ischemia-reperfusion injury, leading to oxidative stress and an increase in reactive oxygen species (ROS) and affecting the vascular endothelial regulatory system via NO-mediated pathways. Chronic intermittent hypoxia can cause a systemic inflammatory response, exacerbating vascular damage and atherosclerosis. In recent years, oxidative stress and inflammatory response have been considered important mechanisms causing cardiovascular and cerebrovascular pathologies in OSA (36).

Stratified analysis by ESS score showed that the incidence of cerebrovascular disease in patients with ESS score < 9 had a significant with ESS score in the NoSAS risk stratification, however, the adjusted model showed that NoSAS risk stratification was not significantly associated with the incidence of cerebrovascular disease, suggesting that the association between OSA evaluated by NoSAS and cerebrovascular disease in patients without symptoms of sleepiness may be a chance finding due to the of multiple comparisons or limited statistical power. Easily overlooked, high NoSAS scores may suggest the need for active intervention (e.g., CPAP) to prevent cerebrovascular events. Stratification by age and gender did not reach statistical significance, possibly because cerebrovascular disease is driven by multiple factors (e.g., atherosclerosis, atrial fibrillation), masking the independent contribution of OSA (as assessed by NoSAS), and higher-quality trials are needed in the future to further validate the data.

## 5 Limitations

Although this study adopted a prospective cohort design, the following limitations remain: (1) The identification of cerebrovascular events was primarily based on patient self-reports and existing clinical records, as systematic neuroimaging examinations were not routinely performed. (2) Although the combination of medical record review and telephone follow-up maximized data completeness and accuracy, misclassification of events may still occur. (3) Due to the lack of comparison with the gold standard polysomnography (PSG), participants with NoSAS scores below the risk threshold may represent false-negative cases. (4) Additionally, some events occurred outside the participating centers, making it impossible to obtain complete clinical records. These limitations underscore the need for future prospective studies to incorporate standardized objective diagnostic validation to further confirm the prognostic value of the NoSAS score. Concurrently, future confirmatory studies should stratify sampling for PSG examinations in screening-negative individuals whenever possible to accurately assess the reliability of this score in OSA diagnosis.

## 6 Conclusion

The NoSAS high-risk group showed a higher incidence of cerebrovascular disease, which could be used as an independent predictor of the disease and may have higher predictive value in the high-risk group of non-sleepy OSA.

## Data Availability

The original contributions presented in the study are included in the article/supplementary material, further inquiries can be directed to the corresponding authors.
